# Sensory acceptance of four flavors of fish-fortified instant porridge for complementary feeding of infants and young children

**DOI:** 10.3389/fnut.2026.1839118

**Published:** 2026-08-12

**Authors:** Kathleen Ragsdale, Terezie Tolar-Peterson, Kendra A. Byrd, Netsayi N. Mudege, Sylvia C. Banda

**Affiliations:** 1 Social Science Research Center, Mississippi State University, Mississippi State, MS, United States; 2 College of Health Sciences, Touro University California, Vallejo, CA, United States; 3 Department of Food Science and Nutrition, California Polytechnic State University, San Luis Obispo, CA, United States; 4 WorldFish, Lusaka Office, Lusaka, Zambia; 5 Sylva Food Solutions, Lusaka, Zambia

**Keywords:** ComFA+fish, complementary feeding, complementary food for Africa+fish powder, fish powder, fish-fortified complementary foods, infant and young child malnutrition

## Abstract

**Background:**

Porridges fortified with locally available, nutrient rich foods can increase nutrient intakes among infants and young children (IYC), but the addition of ingredients such as fish powder may reduce acceptability. We conducted separate sensory panels among adults and caregiver/IYC pairs in rural Zambia to evaluate the acceptability of four fish-fortified complementary instant porridges [strawberry, vanilla, plain, and vanilla+extra fish powder (EFP)], a product we call ComFA+Fish (Complementary Food for Africa+Fish Powder). We calculated ComFA+Fish’s contribution to daily macronutrient requirements for IYC ages 6–23 months, and compared its macronutrient content to three commercially available complementary porridges common to sub-Saharan Africa (SSA).

**Methods:**

Using a five-point hedonic scale, we evaluated porridge acceptability among adults (*N* = 53) and caregiver/IYC pairs (*N* = 33). Adults evaluated each porridge on five sensory attributes, convenience, and overall acceptability; caregivers evaluated their child’s global liking based on observed feeding behaviors. Differences in acceptability across the four flavors were assessed using the Friedman test.

**Results:**

Sensory acceptability was high across both panels. Among adults, strawberry scored significantly higher than all other flavors on average sensory score; however, for convenience and overall acceptability, only strawberry and vanilla+EFP differed significantly, suggesting broad acceptance of the remaining three flavors. Among IYC, strawberry was rated significantly higher than vanilla+EFP on global liking, with no other significant pairwise differences. The proposed daily serving sizes met 24–83% of the recommended protein intakes across the three age groups (6–8, 9–11, and 12–23 months). The plain porridge’s macronutrient profile was similar to or exceeded that of three commercially available complementary porridges common to SSA.

**Conclusion:**

These findings support the viability and acceptability of four ComFA+Fish flavors as a culturally appropriate, locally sourced solution to address IYC malnutrition in rural Zambia, with strong potential for scaling across sub-Saharan Africa.

## 1 Introduction

**Figure 1:**
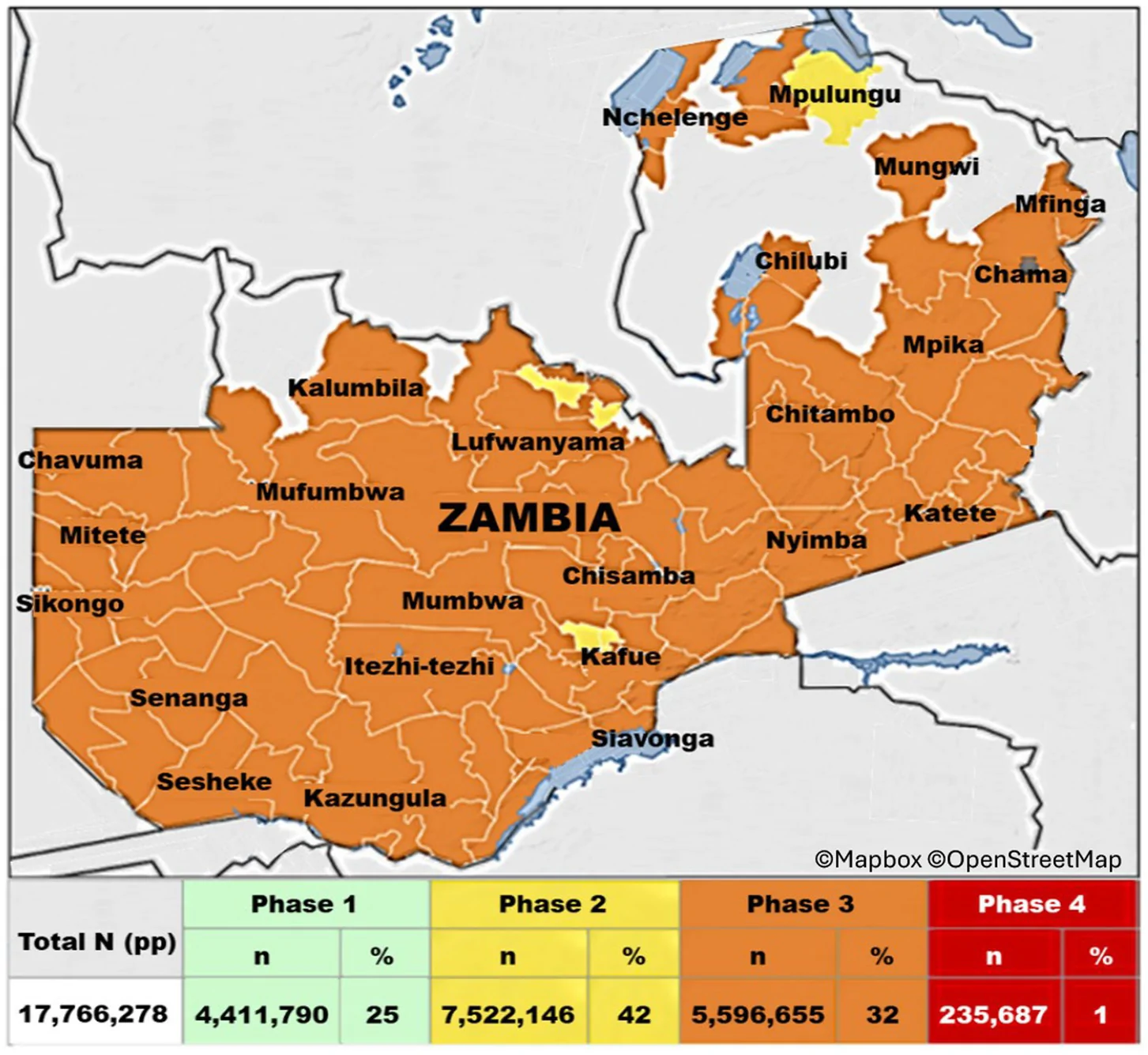
Zambia’s projected acute food insecurity (IPC Phase 3 or above). Open source: IPC Global Partnership: https://www.ipcinfo.org/ipc-country-analysis/details-map/en/c/1157977/?iso3=ZMB.

Child malnutrition contributes to 45% of deaths in children under five annually and leads to impaired cognitive development in 250 million, mostly in low- and middle-income countries (LMIC) (1–5). The U. S. Agency for International Development (USAID) and other key humanitarian donors have helped to dramatically reduce childhood mortality in LMIC. However, the abrupt closure of USAID in early 2025 and the sudden withdrawal of this agency’s humanitarian support is expected to severely increase malnutrition among vulnerable IYC in LMIC as it remains unknown how other donors will fill the gap left by USAID (6–8). It is well-documented that the impacts of malnutrition in childhood “amplify across the life course” (9). This results in a major health burden for LMIC such as Zambia, where 35% of children under five are stunted and 4% are wasted (10–12). According to projections by the Global Partnership of the Integrated Food Security Phase Classification (IPC Global Partnership), 33% of Zambia’s population face acute food insecurity (IPC Phase 3 or higher) and rural areas will be impacted most severely (Figure 1) (13).

Within LMIC families experiencing food insecurity, IYC that have begun the transition from consuming only breastmilk to eating solid foods (i.e., from ages 6–23 months) are at increased nutritional vulnerability as compared to infants who have yet to transition from consuming only breast milk. This nutritional vulnerability is linked to the fact that typical “weaning foods” in food-insecure families are often low-cost porridges that the entire family already consumes, which are carbohydrate-rich yet low in nutrients. Due to the increased nutritional vulnerability of at-risk IYC, the complementary feeding stage is a key intervention point for addressing childhood malnutrition. However, many of such interventions are exclusively cereal-based and/or their impacts on IYC malnutrition are ambiguous due to evidence quality (14). Recent systematic reviews suggest that access to affordable complementary foods for at-risk IYC in many LMIC is lacking, particularly nutrient-rich animal-source foods (15–18).

For example, Ryckman et al. found that at-risk IYC lacked affordable sources of zinc, while affordable sources of iron, calcium, and animal-source foods were scarce in Ethiopia, Mozambique, South Africa, Tanzania, Uganda, and Zambia (15). Similarly, Ryckman et al. found no affordable zinc sources for at-risk IYC in Bangladesh, India, and Pakistan, alongside a lack of affordable iron and calcium sources across all three countries and limited animal-source food sources in Bangladesh (16). Akalu et al. found low consumption of iron-rich/animal-source foods and high consumption of tubers and cereals among IYC across 35 sub-Saharan countries (17). Bailey et al. found that the global distribution of micronutrient deficiencies is widespread for iron, iodine, folate, vitamin A and zinc and that most vulnerable IYC experience multiple deficiencies (18). Furthermore, recent systematic reviews of multiple micronutrient powders (MNP)—globally standardized prepackaged vitamin/mineral sachets distributed by donors such as the United Nations International Children’s Emergency Fund (UNICEF)—have found that MNP have helped reduce global rates of anemia but have had less impact on IYC growth (19–23).

Efforts have increased to shift reliance on imported products to locally sourced solutions to address malnutrition among food insecure IYC in LMIC such as Zambia. Recent disruptions to global humanitarian aid have further highlighted the need for such locally sourced solutions. An important part of this shift has focused on fortifying cereal-based complementary foods with locally sourced fish powder made from protein-rich small pelagic fish. These efforts are feasible as whole small pelagic fish (both fresh and dried) and fish powder are staple foods widely consumed across sub-Saharan Africa (SSA) (24–29). These tiny fish are consumed whole—including the gut, brain, and eyes—meaning that their nutrient concentration is often higher than that of a fish fillet alone. Additionally, the process of drying small pelagic fish concentrates their nutritional content. As a result, fish powder made from small pelagic fish provides a relatively large amount of macro- and micronutrients—even when consumed in the small quantities that accommodate IYC’s tiny gastric systems—while providing nutrients including protein, lipids (e.g., essential fats and fat-soluble vitamins such as vitamins A, D, E, and K), zinc, and vitamin B12 (30–33).

When used to fortify high-carbohydrate/low-protein complementary porridge consumed—of necessity—by many IYC living in poverty, locally sourced fish powder can substantially increase the nutrient concentration this staple food. Yet, despite being widely consumed across Zambia and SSA, locally sourced fish powder is largely underutilized to fortify complementary porridge. Women and community health workers in rural Zambia report that a barrier to uptake of home-fortified complementary porridge is that dehydrating and preparing all the ingredients separately takes time that many caregivers simply do not have (28). Further, the acceptance rate of infants, young children, and their caregivers of novel flavors of fish-fortified complementary foods is largely unknown. Therefore, the aim of this study was to assess the viability (overall acceptability and perceived convenience among adults) and global liking and related variables (among IYC) of four fish-fortified complementary instant porridges, and to determine the flavor that is most accepted of the four.

## 2 Materials and methods

### 2.1 Product formulation

**Figure 2:**
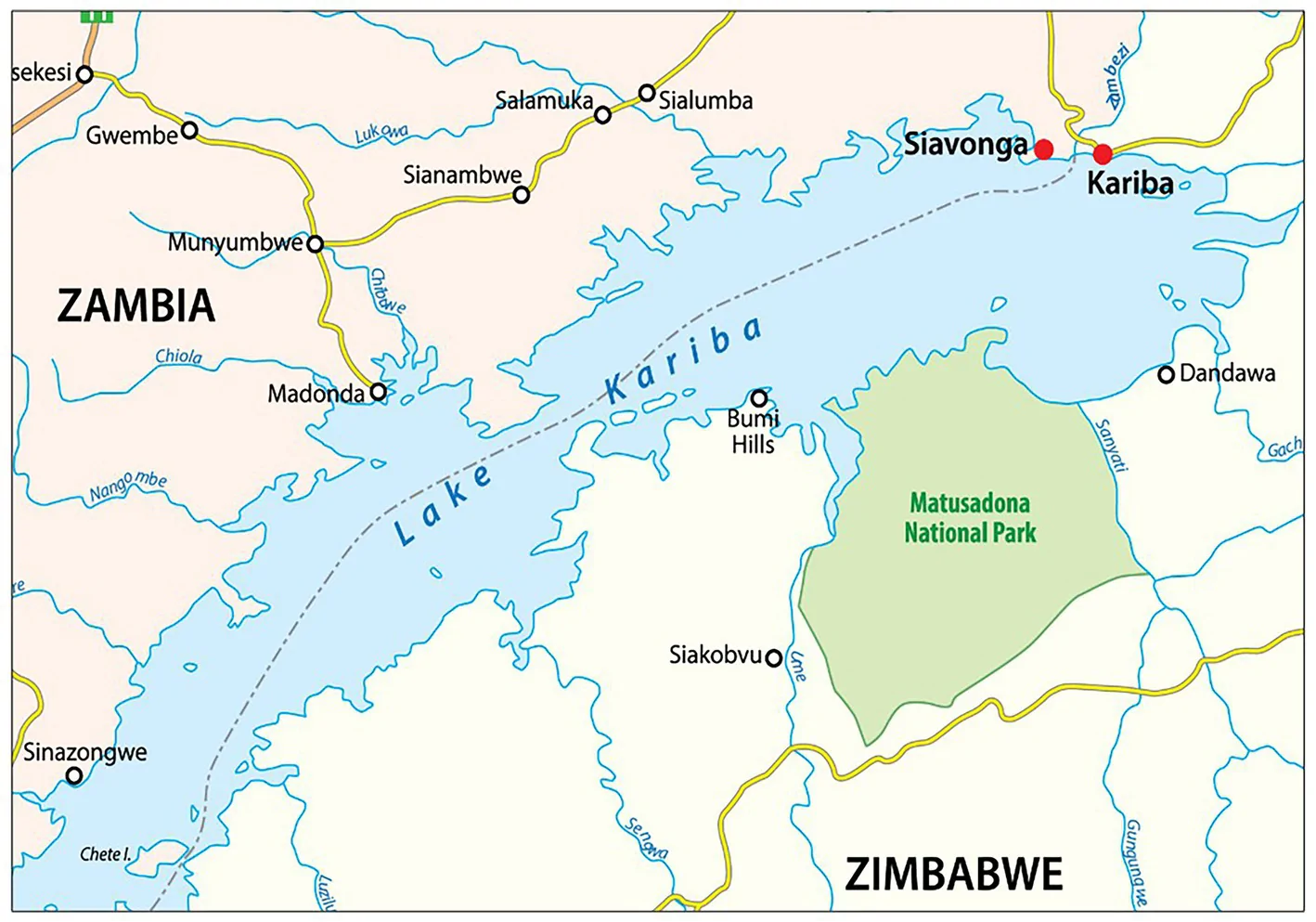
Map of Zambia’s Lake Kariba, which is a major producer of *kapenta* (*Limnothrissa miodon* and *Stolothrissa tanganicae*), showing the Siavonga study site. Source: K. Ragsdale, Mississippi State University.

Initial activities in preparation for the present study included conducting a nutrient analysis of fish powder made from small pelagic fish (*Limnothrissa miodon* and *Stolothrissa tanganicae*, locally known as *kapenta*) sourced from Lake Kariba (Figure 2), which is a major kapenta producer, and conducting two sets of sensory panels. These panels confirmed acceptability of fish-fortified complementary porridge among adults and IYC (34). Next, we partnered with Sylva Food Solutions—a Zambian processing and marketing company whose facility in Lusaka specializes in using locally sourced foods to produce pre-packaged products for Zambian and European markets—to produce two fish-fortified complementary instant porridges (plain and vanilla) using *L. miodon* and *S. tanganicae* for a third sensory panel that confirmed high acceptability of both porridges (35).

For the present study, we partnered with Sylva Food Solutions to create four flavors of fish-fortified instant porridges that again utilized *L. miodon* and *S. tanganicae*. The four flavors included strawberry, vanilla, plain, and vanilla+extra fish powder (EFP) and were evaluated during separate sensory panels among adults and among caregiver/IYC pairs to determine which specific porridges were most acceptable to both adults and IYC and, therefore, had the widest potential for scaling. The standard ingredients of all four flavors included fish powder, provitamin A-biofortified maize meal, soybean flour, sorghum flour, millet flour, baobab flour, moringa flour, sugar, and salt. Supplementary Appendix 1 includes the ingredients of each flavor of porridge, including their amounts. The instant porridge is designed to be sold in individual sachets of 50 gram (g), which preserves the product’s freshness in humid/high-temperature environments that may lack access to reliable refrigeration, protects the product from contamination, and is more likely to be affordable for resource-limited consumers. When preparing a sachet of the instant porridge for serving, the recommended amount of liquid per sachet is 5.65 ounces (167 mL) of water per one 50 g sachet.

### 2.2 Recruitment and eligibility

Participants were recruited from rural fishing communities in Siavonga District, which is located on Lake Kariba approximately 195 km from Zambia’s capital city of Lusaka (Figure 2). The IYC sensory panel was conducted among a purposive sample of caregiver/IYC pairs. Eligible IYC were: (1) 6–23 months old, (2) had no known allergy to fish powder, maize, soybean, sorghum, or millet, and (3) had consumed fish and/or fish-based foods and maize porridge prior to recruitment.

The adult sensory panel was conducted among a purposive sample of men and women who were: (1) 18 years of age or older, (2) had no known allergy to any of the porridge ingredients (3) had consumed fish and/or fish-based foods and maize porridge prior to recruitment, and (4) self-identified as community leaders, decision-makers over their household’s food consumption, and/or fishers, fish processors, and fish sellers. We purposively recruited men to participate in two trainings and the adults’ sensory panel as our previous scaling exercise conducted at Lake Kariba indicated that “in their traditional roles as husbands and fathers, [men] typically exert great decision-making control over household consumption,” yet have far less knowledge of human nutrition in general, and of the specific dietary needs of IYC in particular, as compared to women (28). The scaling exercise participants noted that knowledge of kapenta’s high nutritional value is uncommon among male fishers and, therefore, men often sell their entire kapenta catch at the landing site, which “leaves no fish for the family” (28).

The study protocol was approved by the Mississippi State University Institutional Review Board (IRB No. 24–298). Participation was voluntary and adults provided written informed consent, including consent to be video/audio recorded and photographed. Caregivers provided written parental permission, including permission for their child to be video/audio recorded and photographed. Adults were provided with meal/travel stipends to offset costs associated with participation. Caregivers were provided with meal/travel stipends, lodging for two nights, and childcare products (e.g., diapers, baby wipes, hand sanitizer) to offset costs associated with participation.

### 2.3 Adult sensory panel

**Figure 3:**
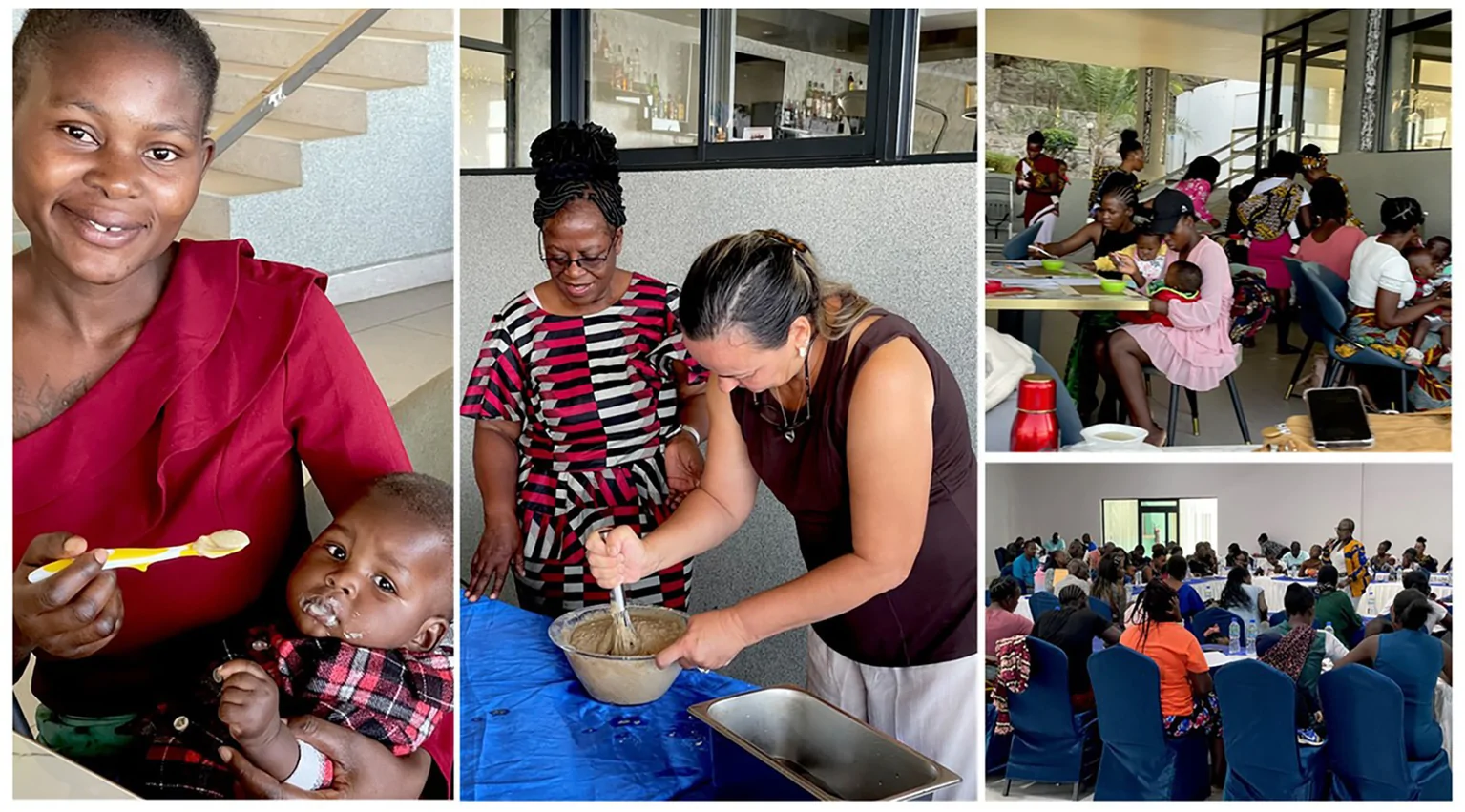
Left: A caregiver and her infant during a sensory panel. Middle: Research team members, Sylvia Banda (left, Sylva Food Solutions) and Terezie Tolar-Peterson (right, Touro University California), prepare a batch of instant porridge for a sensory panel. Top Right: Caregiver/infant pairs participate in a sensory panel. Bottom Right: Adults participate in the nutrition and water, sanitation, and hygiene (WASH) workshop. Photography credit: K. Ragsdale, Mississippi State University.

On day one, adults attended a nutrition/water, sanitation, and hygiene (WASH) workshop (Figure 3) and evaluated the plain instant porridge during a mid-morning break and the vanilla instant porridge during a mid-afternoon break. On day two, adults attended a fish processing/food safety/WASH workshop and evaluated the vanilla+EFP instant porridge during a mid-morning break and the strawberry instant porridge during a mid-afternoon break. For the adults’ evaluations, we implemented the same protocol successfully implemented during two previous sensory panels reported elsewhere (34, 35). This protocol’s development was informed by Madrelle et al. and others (36–39) and included simplified versions of the technical definitions of seven attributes used to evaluate each porridge (e.g., aroma, appearance, texture, taste, sweetness, convenience, and overall acceptability). Participants were not informed of the porridge’s flavor before being served, were served 50 g (∼8 ounces) of porridge in identical 8-ounce BPA-free bowls and were provided with identical small spoons. For each evaluation, participants were instructed to eat freely and to complete their evaluation using the provided form (Supplementary Appendix 2) to evaluate the porridge on seven attributes using a five-point hedonic scale where 1 = extremely disliked, 3 = neither liked nor disliked, and 5 = extremely liked. The evaluation form included simple “smiley face” images corresponding to each point on the five-point scale, and the adults were instructed to mark the appropriate smiley face with an “x.”

### 2.4 IYC sensory panel

On day one, caregivers evaluated whether their IYC found the plain instant porridge acceptable (at breakfast), attended the nutrition/WASH workshop, and evaluated whether their child found the vanilla instant porridge acceptable (during a mid-afternoon break) (Figure 3). On day two, caregivers evaluated whether their IYC found the vanilla+EFP instant porridge acceptable (at breakfast), attended the fish processing/food safety/WASH workshop, and evaluated whether their IYC found the strawberry instant porridge acceptable (during a mid-afternoon break). For the IYC sensory panel, we implemented the same protocol successfully implemented during a previous IYC sensory panel reported elsewhere (34). The protocol’s development was informed by Madrelle et al. and others (37–39). Caregivers used the provided evaluation form (Supplementary Appendix 3) to answer four items: (1) child’s global liking of that porridge, (2) child’s actual consumption of that porridge, (3) child’s relative consumption of that porridge vis-à-vis what the caregiver would expect their child to consume during a typical feeding at that time of day, and (4) the number of full servings of that porridge that their child consumed.

#### 2.4.1 IYC’s global liking

Caregivers were instructed to use a set of five descriptors of common positive/negative feeding behaviors exhibited by their child to evaluate how much their child enjoyed that specific porridge (e.g., opening the mouth to accept the offered food, turning the face away from the offered food). The five descriptors correlated with a five-point hedonic scale where 1 = extremely disliked, 3 = neither liked nor disliked, and 5 = extremely liked. The evaluation form included simple “smiley face” images corresponding to each point on the five-point scale, and caregivers were instructed to mark the appropriate smiley face with an “x.” For item one, caregivers fed the first three spoonfuls of porridge to their child at Time 1 (T1) and selected the appropriate smiley face that best corresponded with how well their child liked the porridge at T1. Caregivers next fed the second three spoonfuls of porridge to their child at Time 2 (T2) and selected the appropriate smiley face that best corresponded with how well their child liked the porridge at T2. Finally, caregivers fed the third three spoonfuls of porridge to their child at Time 3 (T3) and selected the appropriate smiley face that best corresponded with how well their child liked the porridge at T3. Caregivers were allowed to offer their child small sips of water during T1-T3.

#### 2.4.2 IYC’s actual consumption

Caregivers estimated the actual amount of porridge that their child consumed by summing T1 + T2 + T3. Caregivers then used a five-point scale to record their child’s porridge consumption, where 1 = child consumed less than 1/4 of the serving, 3 = child consumed about 1/2 of the serving, and 5 = child consumed the entire serving. The evaluation form included a simple set of black-and-white “bowl” images corresponding to each point on the five-point scale, and caregivers were instructed to mark the appropriate bowl image with an “x.” For example, if the caregiver determined that their child had consumed the entire serving, the caregiver selected the uncropped image of the bowl labeled “entire portion.” And if the caregiver determined that their child consumed ~3/4 of the serving, the caregiver selected the slightly cropped bowl image labeled “about 3/4.”

#### 2.4.3 IYC’s relative consumption

Caregivers were asked to compare their response to item two (i.e., their child’s actual consumption) with what they would expect their child to consume during a typical feeding at that time of day (e.g., breakfast). For this item, we used the same five-point scale, black-and-white “bowl” images, and corresponding label for each bowl image that was used for item two of the evaluation form.

#### 2.4.4 Number of full servings IYC consumed

After completing the first three items, caregivers were allowed to offer their child as many additional servings of porridge as the child wanted. Once the caregiver determined that their child was satiated, the caregiver completed item four. For this item, caregivers were instructed to: (1) quantify how many full servings of that specific porridge their child consumed and (2) complete the “fill in the blank” item to describe any issue or circumstance that might be relevant to their child’s appetite during that evaluation (e.g., the child was breastfed just prior to the evaluation).

### 2.5 Statistical analysis

The mean and SD scores for each sensory characteristic reported by adults on the 5-point hedonic scale were calculated. An overall mean and SD based on the five sensory characteristics was calculated (the average sensory score). A Shapiro–Wilk test (*p* < 0.05) was used to assess normality for the three main outcomes: average sensory score, convenience rating, and acceptability rating. Given the data were not normally distributed for all flavors, we assessed the differences in the outcomes by flavor using the Friedman test. The mean and SD global liking scores of the 5-point hedonic scale, as provided by the caregivers of the IYC, were averaged across the three time points for each IYC. The Friedman test was used for analysis of infant global liking, as Shapiro–Wilk tests indicated non-normal distributions across all four flavors (all *p* < 0.05). A Spearman rank correlation was conducted to examine whether infant age was associated with acceptance scores. No significant relationship was found (rho = 0.07, *p* = 0.474), therefore age in months was not included as a covariate. For both IYC and adults, where the Friedman test indicated significant differences, pairwise post-hoc comparisons were conducted using the Nemenyi-Wilcoxon-Wilcox all-pairs test for a two-way balanced complete block design, with *p*-values adjusted using the single-step method to control for multiple comparisons (40). All analyses were conducted in R (version 4.4.2) using the PMCMRplus package. Differences were considered significant at the level of *p* < 0.05.

### 2.6 Macronutrient analysis

A vacuum-sealed 100 g sample of the plain instant porridge was provided by Sylva Food Solutions to WorldFish in Lusaka, which stored the sample at ambient temperatures prior to shipping to Microchem Specialized Lab Services (Microchem), a South African National Accreditation System (SANAS)-certified laboratory (T0393) in Johannesburg, South Africa, for nutrient analysis. All analyses were conducted using Microchem’s accredited standard operating procedures (S. O. P. C.). Results are reported per 100 g of sample as received. We also provide a comparison of the macronutrient content per 100 g of the plain instant porridge and three commercial complementary porridge powders broadly available in SSA.

## 3 Results

### 3.1 Adult sensory panel

Of the 53 adults invited, 51 had complete data for the strawberry, vanilla, and plain sensory panels, and 50 completed the vanilla+EFP panel. The mean age was 31.8 (SD 10.1) years (range: 18–70), 34 participants were women and 19 were men. The average scores for all sensory attributes by flavor are presented in Tables 1–4. The only attribute mean score that fell below 4.0 was taste for the vanilla+EFP flavor (mean = 3.8, Table 4), meaning all other attributes across all four flavors fell within the “liked” or “extremely liked” range. Five participants were excluded from statistical analysis due to incomplete data, yielding a final sample of 46 for the Friedman test. Strawberry scored significantly higher than the other three flavors on average sensory score (mean = 4.80 vs. 4.41, 4.30, and 4.04 for vanilla, plain, and vanilla+EFP, respectively), which did not differ significantly from each other (Table 5). For both convenience and overall acceptability, the only significant difference was between strawberry and vanilla+EFP. Notably, no significant difference in acceptability was found between vanilla and vanilla+EFP (*p* = 0.188), suggesting that the addition of extra fish powder did not adversely affect consumer acceptance of the vanilla-flavored product.

**Table 1:** Adults’ sensory panel: strawberry instant porridge results (*N* = 51).

Sensory attributes	Extremely liked % (*n*)	Liked % (*n*)	Neutral % (*n*)	Disliked % (*n*)	Extremely disliked % (*n*)	Mean (SD)
Aroma	82.4 (42)	15.7 (8)	2 (1)	–	–	4.80 (0.45)
Appearance	88.2 (45)	9.8 (5)	2 (1)	–	–	4.86 (0.40)
Texture	84.3 (43)	15.7 (8)	—	–	–	4.84 (0.37)
Taste	84.3 (43)	13.7 (7)	2 (1)	–	–	4.82 (0.43)
Sweetness	72.5 (37)	19.6 (10)	7.8 (4)	–	–	4.65 (0.63)

**Table 2:** Adults’ sensory panel: vanilla instant porridge results (*N* = 51).

Sensory attributes	Extremely liked % (*n*)	Liked % (*n*)	Neutral % (*n*)	Disliked % (*n*)	Extremely disliked % (*n*)	Mean (SD)
Aroma	58.8 (30)	31.4 (16)	9.8 (5)	–	–	4.49 (0.67)
Appearance	64.7 (33)	25.5 (13)	9.8 (5)	–	–	4.55 (0.67)
Texture	45.1 (23)	45.1 (23)	7.8 (4)	2 (1)	–	4.33 (0.71)
Taste	53.1 (26)	32.7 (16)	10.2 (5)	4.1 (2)	–	4.35 (0.83)
Sweetness	54.9 (28)	31.4 (16)	7.8 (4)	5.9 (3)	–	4.35 (0.87)

**Table 3:** Adults’ sensory panel: plain instant porridge results (*N* = 51).

Sensory attributes	Extremely liked % (*n*)	Liked % (*n*)	Neutral % (*n*)	Disliked % (*n*)	Extremely disliked % (*n*)	Mean (SD)
Aroma	56.6 (30)	24.5 (13)	9.4 (5)	9.4 (5)	–	4.2 (0.99)
Appearance	65.4 (34)	30.8 (16)	3.8 (2)	–	–	4.6 (0.57)
Texture	42.3 (22)	48.1 (25)	5.8 (3)	3.8 (2)	–	4.3 (0.75)
Taste	46.2 (24)	34.6 (18)	11.5 (6)	7.7 (4)	–	4.3 (0.92)
Sweetness	50.9 (27)	34 (18)	13.2 (7)	1.9 (1)	–	4.3 (0.85)

**Table 4:** Adults’ sensory panel: vanilla+EFP instant porridge results (*N* = 50).

Sensory attributes	Extremely liked % (*n*)	Liked % (*n*)	Neutral % (*n*)	Disliked % (*n*)	Extremely disliked % (*n*)	Mean (SD)
Aroma	44 (22)	30 (15)	14 (7)	12 (6)	–	4.06 (1.04)
Appearance	36 (18)	44 (22)	10 (5)	8 (4)	2 (1)	4.40 (0.99)
Texture	38 (19)	36 (18)	20 (10)	6 (3)	–	4.06 (0.91)
Taste	32.7 (16)	34.7 (17)	14.3 (7)	16.3 (8)	2 (1)	3.80 (1.14)
Sweetness	52 (26)	26 (13)	16 (8)	6 (3)	–	4.24 (0.94)

**Table 5:** Average scores (mean and SD) by flavor as reported by adult participants and differences in scores using the Friedman test (*n* = 46).

Flavor	Average sensory score	Convenience rating	Overall acceptability
Strawberry	4.80 (0.39)^a^	4.82 (0.43)^a^	4.82 (0.43)^a^
Vanilla	4.41 (0.63)^b^	4.45 (0.90)^ab^	4.55 (0.58)^ab^
Plain	4.3 (0.61)^b^	4.5 (0.75)^ab^	4.4 (0.75)^ab^
Vanilla+Extra Fish Powder (EFP)	4.04 (0.78)^b^	4.20 (1.03)^b^	4.12 (0.94)^b^

### 3.2 IYC sensory panel

Of the 33 IYC invited to participate with their caregivers, the mean age was 14.3 (SD 6.5) months (range: 6–23 months). 42.4% were aged 6–11 months (*n* = 14), and 63.6% were female (*n* = 21). Seven participants were excluded from statistical analysis due to incomplete data across flavor conditions, yielding a final sample of 26 for the Friedman test. For each child, global liking scores were averaged across T1, T2, and T3 to yield a single mean global liking score per flavor of porridge; these averaged scores are included in Tables 6–9. They are summarized by flavor in Table 10 and served as the input for the Friedman test. Strawberry ranked significantly higher on global liking than vanilla+EFP (mean = 4.64 vs. 4.01); no other pairwise differences were significant, including between strawberry, vanilla (mean = 4.53), and plain (mean = 4.15) (Table 10).

**Table 6:** IYC sensory panel: strawberry instant porridge results for global liking, actual porridge consumption, relative porridge consumption, and total number of full servings consumed.

Child’s global liking of their porridge serving at Time 1-Time 3 (T1-T3)	Extremely liked % (*n*)	Liked % (*n*)	Neutral % (*n*)	Disliked % (*n*)	Extremely disliked % (*n*)
T1 (*n* = 29)	79.3 (23)	17.2 (5)	–	–	3.4 (1)
T2 (*n* = 29)	79.3 (23)	17.2 (5)	–	–	3.4 (1)
T3 (*n* = 26)	73.1 (19)	23.1 (6)	–	–	3.8 (1)
Child’s actual consumption of their porridge serving (*n* = 28)	Consumed full serving % (*n*)	Consumed ¾ serving % (*n*)	Consumed ½ serving % (*n*)	Consumed ¼ serving % (*n*)	Consumed <¼ serving % (*n*)
T1 + T2 + T3	53.6 (15)	17.9 (5)	3.6 (1)	10.7 (3)	14.3 (4)
Child’s relative consumption of their porridge serving (*n* = 28)	Consumed usual amount % (*n*)	Consumed ¾ usual amount % (*n*)	Consumed ½ usual amount % (*n*)	Consumed ¼ usual amount % (*n*)	Consumed <¼ usual amount % (*n*)
T1 + T2 + T3 vs. child’s usual intake	60.7 (17)	10.7 (3)	3.6 (1)	10.7 (3)	14.3 (4)
No. of full servings the child consumed (*n* = 16)	3 servings % (*n*)	2 servings % (*n*)	1.5 servings % (*n*)	1 serving % (*n*)	<1 serving % (*n*)
T1 + T2 + T3	–	18.8 (3)	–	50 (8)	31.3 (5)

**Table 7:** IYC sensory panel: vanilla instant porridge results for global liking, actual porridge consumption, relative porridge consumption, and total number of full servings consumed.

Child’s global liking of their porridge serving at Time 1-Time 3 (T1-T3)	Extremely liked % (*n*)	Liked % (*n*)	Neutral % (*n*)	Disliked % (*n*)	Extremely disliked % (*n*)
T1 (*N* = 33)	69.7 (23)	15.2 (5)	6.1 (2)	3 (1)	6.1 (2)
T2 (*N* = 33)	66.7 (22)	21.2 (7)	3 (1)	3 (1)	6.1 (2)
T3 (*n* = 31)	64.5 (20)	25.8 (8)	3.2 (1)	–	6.5 (2)
Child’s actual consumption of their porridge serving (*N* = 33)	Consumed full serving % (*n*)	Consumed ¾ serving % (*n*)	Consumed ½ serving % (*n*)	Consumed ¼ serving % (*n*)	Consumed <¼ serving % (*n*)
T1 + T2 + T3	57.6 (19)	12.1 (4)	9.1 (3)	12.1 (4)	9.1 (3)
Child’s relative consumption of their porridge serving (*n* = 31)	Consumed usual amount % (*n*)	Consumed ¾ usual amount % (*n*)	Consumed ½ usual amount % (*n*)	Consumed ¼ usual amount % (*n*)	Consumed <¼ usual amount% (*n*)
T1 + T2 + T3 vs. child’s usual intake	67.7 (21)	6.5 (2)	6.5 (2)	9.7 (3)	9.7 (3)
No. of full servings the child consumed (*n* = 17)	3 servings % (*n*)	2 servings % (*n*)	1.5 servings % (*n*)	1 serving % (*n*)	<1 serving % (*n*)
T1 + T2 + T3	11.8 (2)	11.8 (2)	–	64.7 (11)	11.8 (2)

**Table 8:** IYC sensory panel: plain instant porridge results for global liking, actual porridge consumption, relative porridge consumption, and total number of full servings consumed.

Child’s global liking of their porridge serving at Time 1-Time 3 (T1-T3)	Extremely liked % (*n*)	Liked % (*n*)	Neutral % (*n*)	Disliked % (*n*)	Extremely disliked % (*n*)
T1 (*n* = 33)	54.5 (18)	27.3 (9)	3 (1)	6.1 (2)	9.1 (3)
T2 (*n* = 33)	54.5 (18)	27.3 (9)	3 (1)	9.1 (3)	6.1 (2)
T3 (*n* = 32)	46.9 (15)	25 (8)	15.6 (5)	6.3 (2)	6.3 (2)
Child’s actual consumption of their porridge serving (*N* = 33)	Consumed full serving % (*n*)	Consumed ¾ serving % (*n*)	Consumed ½ serving % (*n*)	Consumed ¼ serving % (*n*)	Consumed <¼ serving % (*n*)
T1 + T2 + T3	9.1 (3)	42.4 (14)	24.2 (8)	12.1 (4)	12.1 (4)
Child’s relative consumption of their porridge serving (*N* = 33)	Consumed usual amount % (*n*)	Consumed ¾ usual amount % (*n*)	Consumed ½ usual amount % (*n*)	Consumed ¼ usual amount % (*n*)	Consumed <¼ usual amount % (*n*)
T1 + T2 + T3 vs. child’s usual intake	21.2 (7)	45.5 (15)	15.2 (5)	6.1 (2)	12.1 (4)
No. of full servings the child consumed (*n* = 32)	3 servings % (*n*)	2 servings % (*n*)	1.5 servings % (*n*)	1 serving % (*n*)	<1 serving % (*n*)
T1 + T2 + T3	–	3.1 (1)	–	46.9 (15)	50 (16)

**Table 9:** IYC sensory panel: vanilla+EFP instant porridge results for global liking, actual porridge consumption, relative porridge consumption, and total number of full servings consumed.

Child’s global liking of their porridge serving at Time 1-Time 3 (T1-T3)	Extremely liked % (*n*)	Liked % (*n*)	Neutral % (*n*)	Disliked % (*n*)	Extremely disliked % (*n*)
T1 (*n* = 30)	30 (9)	40 (12)	26.7 (8)	–	3.3 (1)
T2 (*n* = 29)	41.4 (12)	24.1 (7)	20.7 (6)	–	13.8 (4)
T3 (*n* = 29)	41.4 (12)	20.7 (6)	20.7 (6)	10.3 (3)	3.4 (1)
Child’s actual consumption of their porridge serving (*n* = 31)	Consumed full serving % (*n*)	Consumed ¾ serving % (*n*)	Consumed ½ serving % (*n*)	Consumed ¼ serving % (*n*)	Consumed <¼ serving % (*n*)
T1 + T2 + T3	25.8 (8)	6.5 (2)	22.6 (7)	22.6 (7)	22.6 (7)
Child’s relative consumption of their porridge serving (*n* = 27)	Consumed usual amount % (*n*)	Consumed ¾ usual amount % (*n*)	Consumed ½ usual amount % (*n*)	Consumed ¼ usual amount % (*n*)	Consumed <¼ usual amount % (*n*)
T1 + T2 + T3 vs. child’s usual intake	29.6 (8)	7.4 (2)	25.9 (7)	14.8 (4)	22.2 (6)
No. of full servings the child consumed (*n* = 19)	3 servings % (*n*)	2 servings % (*n*)	1.5 servings % (*n*)	1 serving % (*n*)	<1 serving % (*n*)
T1 + T2 + T3	–	15.8 (3)	–	26.3 (5)	57.9 (11)

**Table 10:** IYC mean global liking score (averaged across T1–T3) by flavor as reported by caregivers, and differences in scores using the Friedman test (*n* = 26).

Flavor	Average global liking score
Strawberry	4.64 (0.83)^a^
Vanilla	4.53 (0.96)^ab^
Plain	4.15 (1.12)^ab^
Vanilla+Extra Fish Powder (EFP)	4.01 (1.32)^b^

*Strawberry:* 96.2% of caregivers reported that their child extremely liked/liked the strawberry porridge at T3 (Table 6). Most caregivers (71.4%; *n* = 20) reported that their child consumed 3/4 or more of their serving across the full evaluation period, and 71.4% (*n* = 20) reported consumption at or above their child’s usual intake. The number of full servings consumed ranged from 1 to 2. *Vanilla:* 90.3% of caregivers reported that their child extremely liked/liked the vanilla porridge at T3 (Table 7). Most caregivers (69.7%; *n* = 23) reported their child consumed 3/4 or more of their serving across the full evaluation period, and 74.2% (*n* = 23) reported consumption at or above their child’s usual intake. The number of full servings consumed ranged from 1 to 3. *Plain:* 71.9% of caregivers reported that their child extremely liked/liked the plain porridge at T3 (Table 8). Just over half of caregivers (51.1%; *n* = 17) reported their child consumed 3/4 or more of their serving across the full evaluation period, and 66.7% (*n* = 22) reported consumption at or above their child’s usual intake. The number of full servings consumed ranged from 1–3. *Vanilla+EFP:* The vanilla+EFP porridge showed lower acceptance, although the average global liking was not significantly different than the vanilla or plain flavors (Table 10). 62.1% of caregivers reported that their child extremely liked/liked the vanilla+EFP porridge at T3 (Table 9). Yet less than one-third of caregivers (32.3%; *n* = 10) reported their child consumed 3/4 or more of their serving across the full evaluation period, and only 37% (*n* = 10) reported consumption at or above their child’s usual intake, while 22.2% (*n* = 6) consumed less than 1/4. The number of full servings consumed ranged from 1 to 2.

### 3.3 Macronutrient analysis

The calculations performed reflect the number of sachets per day that would feasibly allow breastmilk and other complementary foods to remain part of the child’s diet. At the 6–8 month age range, complementary foods are recommended two to three times per day. Given that each porridge sachet is 50 g, providing one-half sachet (25 g) once per day is feasible without displacing breastmilk or other diverse complementary foods in the infant’s diet. This half sachet supplies 94 calories, which equals approximately 26% of the recommended caloric needs from complementary foods per day for 6–8 month-olds (41), and 2.7 g of protein, which equals approximately 24% of the recommended 11 g of protein needed per day for 6–8 month-olds (42). The Adequate Intake (AI) established by the Institute of Medicine is 30 g of total fat per day for infants ages 6–12 months (42). Therefore, this half sachet supplies 1.5 g of fat, which equals approximately 5% of the recommended total fat needed per day for 6–8 month-olds (42).

For 9–11 month-olds, as feeding frequency increases to three to four times per day at this age, one sachet (50 g) per day is a feasible amount that still allows room for other diverse complementary foods alongside breastmilk in the infant’s diet. One sachet per day supplies 188 calories, which equals approximately 39% of the recommended caloric needs from complementary foods per day for 9–11 month-olds (41), and 5.4 g of protein, which equals approximately 50% of the recommended 11 g of protein needed per day for 9–11 month-olds (42). As the AI established by the Institute of Medicine is 30 g of total fat per day for infants ages 6–12 months (42), one sachet per day supplies 3.1 g of fat, which equals approximately 10% of the recommended total fat needed per day for 9–11 month-olds (42).

For 12–23 month-olds, two sachets (100 g) per day is feasible while still accommodating other diverse complementary foods in the child’s diet. Two sachets per day supply 377 calories, which equals approximately 50% of the recommended caloric needs from complementary foods for 12–23 month-olds (41), and 10.8 g of protein, which equals approximately 83% of the recommended 13 g of protein needed per day for 12–23 month-olds (42). While the Institute of Medicine has not established an AI for total fat per day for children over 12 months of age, two sachets per day supplies 6.1 g of fat.

### 3.4 Nutrient comparison of ComFA+fish plain instant porridge to three porridges commercially available in SSA

We compared the macronutrient content per 100 g of ComFA+Fish plain instant porridge (Sylva Food Solutions) to three complementary instant porridges broadly commercially available in SSA, using each product’s nutrition label as the basis for that product’s calculation. These complementary porridges included Tombrown (Bliss), Tom Brown (Blue Bay), and Tom Brown PLUS (PachaMama Foods). As Table 11 indicates, all four products were relatively similar in caloric content, but varied more widely in terms of protein and fat content. ComFA+Fish contained 377 calories per 100 g, while the caloric content of all four products ranged from 354 calories for Tom Brown PLUS (PachaMama) to 406 calories for Tom Brown (Blue Bay). ComFA+Fish contained 15.5 g of protein per 100 g, while the protein content of all four products ranged from 7.3 g Tom Brown PLUS (PachaMama) to 24 g for Tombrown (Bliss). ComFA+Fish contained 6.1 g of dietary fat per 100 g, while the fat content of all four products ranged from 2.3 g for Tom Brown (Blue Bay) to 8.7 g for Tombrown (Bliss).

**Table 11:** Macronutrient content per 100 g of ComFA+Fish plain instant porridge flavor compared to three commercially available complementary porridges in sub-Saharan Africa.

Product	Calories	Protein (g/100 g)	Fat (g/100 g)
ComFA+Fish Plain Instant Porridge (Sylva Food Solutions)	377	15.5	6.1
Tom Brown PLUS Porridge (PachaMama Foods)^*^	354	7.3	2.8
Tom Brown Porridge (Blue Bay)^†^	406	21.8	2.3
Tombrown Porridge (Bliss)^‡^	404	24	8.7

*PachaMama Foods: https://pachamamafoodsng.com/product/tom-brown-plus/. ^†^lolagreen.eu: https://www.lolagreen.eu/en/a/tom-brown. ^‡^Bliss: https://www.mynetdiary.com/food/calories-in-tombrown-by-bliss-serving-25189783-0.html.

## 4 Discussion

All four flavors of the fish-fortified complementary instant porridge achieved high sensory acceptability among both the sample of adults and the sample of IYC who participated in sensory evaluations conducted in rural Zambia, with strawberry emerging as the top-ranked flavor across both panels, which is a noteworthy finding given that strawberry and vanilla represent flavor profiles that may be entirely novel to this population. Our inclusion of caregiver/IYC pairs is a meaningful methodological contribution, since adult preferences do not always predict IYC acceptance, and caregiver-reported IYC acceptability is a more proximate indicator of real-world adoption.

The present study’s findings align with a recently published study from Malawi (27), which formulated and evaluated complementary foods developed from blends of maize, soybeans, and locally sourced small pelagic fish (*Engraulicypris sardella*, locally known as *usipa*) from Lake Malawi. That study found that fish-based composite flours had desirable nutritional composition, sensory acceptability, and good shelf-life for household use, and that the addition of fish significantly increased the protein content of the flour mixes, meeting the protein requirement for IYC. This directly mirrors our macronutrient findings, in which ComFA+Fish’s protein content of 15.5 g per 100 g was competitive with or superior to three commercially available complementary porridges common to SSA. Notably, both studies used locally sourced small pelagic fish—kapenta (*Limnothrissa miodon* and *Stolothrissa tanganicae*) from Lake Kariba in our case, and usipa from Lake Malawi in the Malawi study—and both benchmarked their formulations against commercial alternatives, finding their locally sourced fish-fortified products to be nutritionally competitive.

Our findings also resonate with those of Ahern et al., who explored the integration of locally sourced small fish (usipa) fish powder into school meal programs in Malawi among children aged 6–13 years old, which is a population older than the IYC in our study but one that shares the same broader context of nutritional vulnerability in SSA (24). Ahern et al. found that fish powder incorporated into school meal porridge recipes was highly accepted, with approximately 90% of learners consuming over 75% of porridges containing pan-roasted usipa powder. This convergence across age groups and country contexts strengthens the case that fish-fortified porridges are broadly acceptable both during the complementary feeding window and across the life course in SSA.

While the macronutrient analysis demonstrates that ComFA+Fish plain instant porridge can meaningfully contribute to caloric and protein needs across all three age groups, the fat content findings warrant separate consideration. One sachet provides only 1.5–6.1 g of fat depending on the serving size, meeting just 5–10% of the AI of 30 g of total fat per day established by the Institute of Medicine for infants ages 6–12 months (42). This is notable given that dietary fat, and long-chain polyunsaturated fatty acids (LC-PUFAs) in particular, plays a critical role in brain development, myelination, and visual acuity during the first 1,000 days of life (24–26). However, this finding should be interpreted in context. First, breastmilk is itself a rich source of fat and LC-PUFAs, and for breastfed IYC in the 6–11 month age range the fat gap from complementary foods is partially offset. Second, the low-fat content of ComFA+Fish is consistent with the fat content of the commercial comparators in Table 11, two of which (Tom Brown PLUS and Tom Brown Blue Bay) contain even less fat per 100 g. This suggests that low fat content is a sector-wide characteristic of cereal-based complementary porridges in SSA rather than a specific limitation of ComFA+Fish. That said, increasing the fat content of ComFA+Fish at the point of preparation (by adding oil, for example) represents a practical and low-cost strategy to improve the porridge’s fat contribution without requiring reformulation. Future iterations of ComFA+Fish should also explore whether increasing the fish powder content or incorporating alternative locally available fat sources into the formulation could improve the fat profile while maintaining the high sensory acceptability demonstrated in this study.

A deliberate feature of our recruitment strategy was the inclusion of men as participants in the adult sensory panel. As noted in the methods section, men in Lake Kariba fishing communities typically exert significant decision-making control over household food consumption, yet tend to have less knowledge of IYC nutritional needs, and of the high nutritional value of kapenta specifically (28). This dynamic has real consequences for IYC nutrition: when men sell their entire kapenta catch at the landing site without retaining any for household consumption, fish is effectively removed from the family diet. The fact that 19 male participants evaluated all four ComFA+Fish porridges highly is therefore a finding with meaningful practical implications. Men who have directly tasted and positively evaluated a fish-fortified complementary porridge may be more likely to recognize its value as a household food, more inclined to support its purchase, and less likely to prioritize selling fish/fish powder over retaining a portion for family use. In communities where men control household income and purchasing decisions, their buy-in is not a peripheral concern but a prerequisite for sustained adoption of any complementary food product. Future research should explicitly examine whether exposure to and acceptance of ComFA+Fish among male household decision-makers translates into measurable changes in household purchasing behavior, fish allocation decisions, and ultimately IYC dietary quality as this pathway from male sensory acceptance to improved IYC nutrition represents an underexplored but potentially high-impact mechanism for scaling.

### 4.1 Potential limitations

Several limitations should be considered when interpreting these findings. First, both the adult and IYC sensory panels relied on relatively small purposive samples recruited from a single district in rural Zambia, which limits the generalizability of the findings to other communities, regions, or countries. Purposive sampling was appropriate for the exploratory aims of this study, but future research should employ larger, more representative samples to confirm these findings across diverse populations and contexts within SSA. Second, attrition was notable in the IYC panel, where seven participants were excluded from statistical analysis due to incomplete data across flavor conditions, reducing the final analytic sample to 26. While attrition of this kind is common in sensory panel research involving IYC, it reduces statistical power and may have introduced bias if the IYC who did not complete all four tastings differed systematically from those who did. Third, the sensory panels were conducted over two consecutive days, raising the possibility that fatigue, satiation, or carry-over effects influenced participants’ evaluations of the later porridges, particularly the strawberry and vanilla+EFP flavors, which were evaluated on day two. Although this limitation applies equally to both panels, it is particularly relevant for IYC, whose appetite and mood can vary substantially across short time periods. Fourth, the shelf-life of ComFA+Fish was not assessed in this study, which is an important gap given that the target population in rural Zambia may lack reliable refrigeration and that shelf-stability is a prerequisite for scaling. Fifth, this study did not assess the cost of production, affordability to end users, or consumers’ willingness to pay for ComFA+Fish, which are critical determinants of real-world adoption and scaling potential. However, these limitations point to a robust research agenda and should be prioritized in future studies so that feasibility can be more fully assessed.

## 5 Conclusion

The present study contributes to the growing body of evidence that locally sourced fish powder holds promise as a culturally congruent, sustainable, and cost-effective way to fortify complementary instant porridges for vulnerable IYC in LMIC in SSA. While fish powder and the small pelagic fish from which fish powder is produced are widely consumed across SSA, fish powder remains seriously underutilized as a nutrient-rich animal-source food to fortify the cereal-based high carbohydrate/low-nutrient porridges that are staple complementary foods among resource-limited families in this region. Our findings that all four fish-fortified instant porridges were palatable to IYC are highly encouraging, as children’s preferences for certain flavor profiles are linked to higher consumption of certain foods over others. And our findings that all four fish-fortified instant porridges were acceptable to adults are equally encouraging, as adults are both primary caregivers of IYC and are key household decision-makers in terms of the production, purchase, and preparation of foods for IYC and other family members. In a world in which climate change is threatening food production, funneling small pelagic fish from local capture fisheries to vulnerable IYC represents one way of capitalizing on the nutrient supply of a given country. Our findings show both the feasibility and acceptability of using locally available, nutrient-rich fish powder to enhance IYC’s diets in the face of fragile food systems, threats to humanitarian aid, and a highly unstable world.

## Data Availability

The raw data supporting the conclusions of this article will be made available by the authors, without undue reservation.

## References

[1] Black RE Victora CG Walker SP Bhutta ZA Christian P de Onis M . Maternal and child undernutrition and overweight in low-income and middle-income countries. Lancet. (2013) 382:427–51. doi: 10.1016/S0140-6736(13)60937-X 23746772, 23746772

[2] Black MM Walker SP Fernald LCH Andersen CT DiGirolamo A Lu C . Early childhood development coming of age: science through the life course. Lancet. (2017) 389:77–90. doi: 10.1016/S0140-6736(16)31389-7 27717614, 27717614 PMC5884058

[3] United Nations Children’s Fund (UNICEF) Early Childhood Development–UNICEF Vision for Every Child UNICEF (2023). Available online at: https://www.unicef.org/media/145336/file/Early%20Childhood%20Development%20-%20UNICEF%20Vision%20for%20Every%20Child.pdf

[4] World Bank Group Well-Designed Early Childhood Development Programs Can Pay Big Dividends World Bank Group (2017). Available online at: https://www.worldbank.org/en/news/feature/2017/03/30/well-designed-early-childhood-development-programs-can-pay-big-dividends

[5] World Health Organization (WHO) Child Mortality (under 5 Years) WHO (2022). Available online at: https://www.who.int/news-room/fact-sheets/detail/children-reducing-mortality

[6] Cavalcanti DM de Oliveira Ferreira de Sales L da Silva AF Basterra EL Pena D Monti C . Evaluating the impact of two decades of USAID interventions and projecting the effects of defunding on mortality up to 2030: a retrospective impact evaluation and forecasting analysis. Lancet. (2025) 406:283–94. doi: 10.1016/S0140-6736(25)01186-9, 40609560 PMC12274115

[7] Stover J Sonneveldt E Tam Y Horton KC Phillips AN Smith J . Effects of reductions in US foreign assistance on HIV, tuberculosis, family planning, and maternal and child health: a modelling study. Lancet Glob Health. (2025) 13:e1669–80. doi: 10.1016/S2214-109X(25)00281-5 40975076, 40975076 PMC12447089

[8] Anfaal Z Khawar M Iqbal J Rath S. Children at risk: the growing impact of USAID cuts on pediatric malnutrition and death rates. Matern Child Nutr. (2025) 21:e70028. doi: 10.1111/mcn.70028 40097364, 40097364 PMC12150160

[9] Lu C Cuartas J Fink G McCoy D Liu K Li Z . Inequalities in early childhood care and development in low/middle-income countries: 2010-2018. BMJ Glob Health. (2020) 5:e002314. doi: 10.1136/bmjgh-2020-002314, 32133201 PMC7042597

[10] United States Agency for International Development (USAID) Zambia: Nutrition Profile USAID (2021). Available online at: https://www.usaid.gov/sites/default/files/documents/Copy_of_tagged_Zambia-Nutrition-Profile.pdf

[11] UNICEF Country Profiles for Early Childhood Development: Zambia UNICEF (2023). Available online at: https://nurturing-care.org/resources/country-profiles

[12] Zambia Statistics Agency, Ministry of Health, and ICF. Zambia Demographic and Health Survey 2018. Zambia Statistics Agency Ministry of Health ICF (2019). Available online at: https://dhsprogram.com/publications/publication-FR361-DHS-Final-Reports.cfm

[13] Integrated Food Security Phase Classification (IPC) Global Partnership Zambia: Acute Food Insecurity Situation for April–September 2024 and Projection for October 2024–March 2025 IPC Global Partnership (2025). Available online at: https://www.ipcinfo.org/fileadmin/user_upload/ipcinfo/docs/IPC_Zambia_Acute_Food_Insecurity_Jul2024_Mar2025_Report.pdf

[14] Eaton JC Rothpletz-Puglia P Dreker MR Iannotti L Lutter C Kaganda J . Effectiveness of provision of animal-source foods for supporting optimal growth and development in children 6 to 59 months of age. Cochrane Database Syst Rev. (2019) 2019:CD012818. doi: 10.1002/14651858.CD012818.pub2, 30779870 PMC6380771

[15] Ryckman T Beal T Nordhagen S Chimanya K Matji J. Affordability of nutritious foods for complementary feeding in eastern and southern Africa. Nutr Rev. (2021) 79:35–51. doi: 10.1093/nutrit/nuaa137, 33693913 PMC7948081

[16] Ryckman T Beal T Nordhagen S Murira Z Torlesse H. Affordability of nutritious foods for complementary feeding in South Asia. Nutr Rev. (2021) 79:52–68. doi: 10.1093/nutrit/nuaa139, 33693914 PMC7948078

[17] Akalu Y Yeshaw Y Tesema GA Demissie GD Molla MD Muche A . Iron-rich food consumption and associated factors among children aged 6-23 months in sub-Saharan Africa: a multilevel analysis of demographic and health surveys. PLoS One. (2021) 16:e0253221. doi: 10.1371/journal.pone.0253221, 34138916 PMC8211154

[18] Bailey RL West KP Jr Black RE. The epidemiology of global micronutrient deficiencies. Ann Nutr Metab. (2015) 66:22–33. doi: 10.1159/000371618, 26045325

[19] White JM Beal T Arsenault JE Okronipa H Hinnouho G-M Chimanya K . Micronutrient gaps during the complementary feeding period in 6 countries in eastern and southern Africa: a comprehensive nutrient gap assessment. Nutr Rev. (2021) 79:16–25. doi: 10.1093/nutrit/nuaa142, 33693910 PMC7947982

[20] Park JJH Harari O Siden E . Interventions to improve linear growth during complementary feeding period for children aged 6-24 months living in low- and middle-income countries: a systematic review and network meta-analysis. Gates Open Res. (2020) 3:1660. doi: 10.12688/gatesopenres.13083.2, 32259047 PMC7096089

[21] Lassi ZS Padhani ZA Rabbani A Rind F Salam RA das JK . Impact of dietary interventions during pregnancy on maternal, neonatal, and child outcomes in low- and middle-income countries. Nutrients. (2020) 12:531. doi: 10.3390/nu12020531 32092933, 32092933 PMC7071393

[22] Roberts SB Franceschini MA Silver RE Taylor SF de Sa AB Có R . Effects of food supplementation on cognitive function, cerebral blood flow, and nutritional status in young children at risk of undernutrition: randomized controlled trial. BMJ. (2020) 370:m2397. doi: 10.1136/bmj.m2397, 32699176 PMC7374799

[23] De-Regil LM Suchdev PS Vist GE Walleser S Peña-Rosas JP. Home fortification of foods with multiple micronutrient powders for health and nutrition in children under two years of age. Cochrane Database Syst Rev. (2011) 9:CD008959. doi: 10.1002/14651858.CD008959.pub2, 21901727

[24] Ahern M Lowore T Andrianarimanana MH Banda A Toppe J Ng’ong’ola-Manani T. Exploring the integration of fish powder in school meal programs in Malawi through a food environment lens: acceptability, affordability, and convenience. Front Nutr. (2025) 12:1605540. doi: 10.3389/fnut.2025.1605540 40626222 PMC12229799

[25] Ahern M. Mwanza P.S. Genschick S. Thilsted S.H. (2020) Nutrient-Rich Foods to Improve Dietary Quality in the First 1000 Days of Life in Malawi and Zambia: Formulation, Processing and Sensory Evaluation Penang WorldFish. Available online at: https://hdl.handle.net/20.500.12348/4383

[26] Byrd KA Shieh J Mork S Pincus L O'Meara L Atkins M . Fish and fish-based products for nutrition and health in the first 1000 days: a systematic review of the evidence from low and middle-income countries. Adv Nutr. (2022) 13:2458–87. doi: 10.1093/advances/nmac102 36166842, 36166842 PMC9776644

[27] Kaunda G Mwangwela A Geresomo N Kaunda E Simfukwe K. Proximate composition, acceptability, and shelflife of complementary foods developed from blends of staple grains maize, soybeans, and fish in Malawi. Cogent Food Agric. (2025) 11:2544957. doi: 10.1080/23311932.2025.2544957

[28] Mudege N Ragsdale K Kakwasha K Read-Wahidi M Muzungaire L Kolbila R Complementary Food for Africa+Dried fish Powder (ComFA+Fish): Scaling Recommendations. USAID, Feed the Future Innovation Lab for Fish, Mississippi State University (2024). Available online at: http://tinyurl.com/bdd86wp7

[29] Simfukwe K Msukwa AV Mphande J Hasimuna OJ Limuwa MM Kaunda E. Is the concentration of heavy metals in sun-dried *Engraulicypris sardella* (Günther, 1868) in Malawi, a human health risk? J Environ Chem Ecotoxicol. (2024) 6:354–62. doi: 10.1016/j.enceco.2024.08.002

[30] Byrd KA Pincus L Pasqualino MM Muzofa F Cole SM. Dried small fish provide nutrient densities important for the first 1000 days. Matern Child Nutr. (2021) 17:e13192. doi: 10.1111/mcn.13192 33942983, 33942983 PMC8476445

[31] Nölle N Genschick S Schwadorf K Hrenn H Brandner S Biesalski HK. Fish as a source of (micro)nutrients to combat hidden hunger in Zambia. Food Secur. (2020) 12:1385–406. doi: 10.1007/s12571-020-01060-9

[32] Bogard JR Hother AL Saha M Bose S Kabir H Marks GC . Inclusion of small indigenous fish improves nutritional quality during the first 1000 days. Food Nutr Bull. (2015) 36:276289. doi: 10.1177/0379572115598885, 26297705

[33] Haug A Christophersen OA Kinabo J Kaunda W Eik LO. Use of dried kapenta (Limnothrissa miodon and *Stolothrissa tanganicae*) and other products based on whole fish for complementing maize-based diets. Afr J Food Agric Nutr Dev. (2010) 10:2478–500.

[34] Ragsdale K Read-Wahidi MR Mudege NN Iannotti LL Muzungaire L Funduluka P. Sensory panel results of a dried fish powder supplement among caregivers and young children in Zambia. Public Health Nutr. (2023) 27:e32. doi: 10.1017/S1368980023002586, 38031467 PMC10897570

[35] Ragsdale K. Mudege N. Banda S. Read-Wahidi M. R. Muzungaire L. Kolbila R. Complementary Food for Africa+Dried fish Powder (ComFA+Fish) – Sensory panel III Results for two ComFA+Fish Instant Porridges USAID, Feed the Future Innovation Lab for Fish. Social Science Research Center, Mississippi State University (2023). Available online at: https://tinyurl.com/2sejjudx

[36] Madrelle J Lange C Boutrolle I Valade O Weenen H Monnery-Patris S . Development of a new in-home testing method to assess infant food liking. Appetite. (2017) 113:274–83. doi: 10.1016/j.appet.2017.03.002, 28274649

[37] Puri S Rekhi TK Thomas T Jadhav MH Mannar V Diosady LL. Sensory trial of quintuple fortified salt-salt fortified with iodine, iron, folic acid, vitamin B_12_, and zinc-among consumers in New Delhi, India. Food Nutr Bull. (2022) 43:340–50. doi: 10.1177/03795721221078361, 35531896 PMC9403385

[38] Sutrisna A Vossenaar M Izwardy D Tumilowicz A. Sensory evaluation of foods with added micronutrient powder (MNP) “Taburia” to assess acceptability among children aged 6-24 months and their caregivers in Indonesia. Nutrients. (2017) 9:979. doi: 10.3390/nu9090979 32962325, 32962325 PMC5622739

[39] Schwartz C Issanchou S Nicklaus S. Developmental changes in the acceptance of the five basic tastes in the first year of life. Br J Nutr. (2009) 102:1375–85. doi: 10.1017/S0007114509990286 19505346, 19505346

[40] Pohlert T. (2014). The Pairwise Multiple Comparison of Mean Ranks Package (PMCMR). R package. Available online at: https://CRAN.R-project.org/package=PMCMRplus

[41] Dewey KG Brown KH. Update on technical issues concerning complementary feeding of young children in developing countries and implications for intervention programs. Food Nutr Bull. (2003) 24:5–28. doi: 10.1177/156482650302400102 12664525, 12664525

[42] Institute of Medicine of the National Academies (IOM). Dietary Reference Intakes for Energy, Carbohydrate, fiber, fat, Fatty Acids, Cholesterol, Protein and amino Acids 2005. Washington, DC: National Academies Press (2005).

