# Supplementary material for: Sensory acceptance of four flavors of fish-fortified instant porridge for complementary feeding of infants and young children
Source: Front Nutr. 2026 Aug 12;13:1839118. doi: 10.3389/fnut.2026.1839118 (PMC13508812; doi:10.3389/fnut.2026.1839118)
Supplement: Supplementary file 1 [file Table_1.docx]

| Appendix 1. Formulation of ComFA+Fish instant porridge flavors of strawberry, vanilla, plain, and vanilla+extra fish powder (EFP) | | | |
| --- | --- | --- | --- |
| Strawberry instant porridge | Raw  ingredient  (kg) | Raw  ingredient  (%) |  |
| Provitamin A biofortified maize meal | 5.000 | 53.33 |  |
| Kapenta fish powder^*^ | 0.800 | 8.53 |  |
| Moringa powder | 0.150 | 1.60 |  |
| Baobab seed powder | 0.150 | 1.60 |  |
| Soybean flour | 0.800 | 8.53 |  |
| Sorghum flour | 0.750 | 8.00 |  |
| Millet flour | 0.750 | 8.00 |  |
| Sugar | 0.900 | 9.60 |  |
| Salt | 0.075 | 0.80 |  |
| Strawberry flavoring^†^ (Brand: Cape Strawberry) | 0.015 | 0.53 |  |
| Total | **9.375** | **100** |  |
| Vanilla instant porridge |  |  |  |
| Provitamin A biofortified maize meal | 5.000 | 53.53 |  |
| Kapenta fish powder^*^ | 0.800 | 8.57 |  |
| Moringa powder | 0.150 | 1.61 |  |
| Baobab seed powder | 0.150 | 1.61 |  |
| Soybean flour | 0.800 | 8.57 |  |
| Sorghum flour | 0.750 | 8.03 |  |
| Millet flour | 0.750 | 8.03 |  |
| Sugar | 0.900 | 9.64 |  |
| Salt | 0.075 | 0.80 |  |
| Vanilla flavoring^†^ (Brand: Cape Vanilla) | 0.015 | 0.16 |  |
| Total | **9.340** | **100** |  |
| Plain (base) instant porridge |  |  |  |
| Provitamin A biofortified maize meal | 5.000 | 53.62 |  |
| Kapenta fish powder^*^ | 0.800 | 8.58 |  |
| Moringa powder | 0.150 | 1.61 |  |
| Baobab seed powder | 0.150 | 1.61 |  |
| Soybean flour | 0.800 | 8.58 |  |
| Sorghum flour | 0.750 | 8.04 |  |
| Millet flour | 0.750 | 8.04 |  |
| Sugar | 0.900 | 9.65 |  |
| Salt | 0.075 | 0.80 |  |
| Total | **9.325** | **100** |  |
| Vanilla+EFP instant porridge |  |  |  |
| Provitamin A biofortified maize meal | 5.000 | 51.33 |  |
| Kapenta fish powder^*^ | 1.200 | 12.32 |  |
| Moringa powder | 0.150 | 1.54 |  |
| Baobab seed powder | 0.150 | 1.54 |  |
| Soybean flour | 0.800 | 8.21 |  |
| Sorghum flour | 0.750 | 7.70 |  |
| Millet flour | 0.750 | 7.70 |  |
| Sugar | 0.900 | 9.24 |  |
| Salt | 0.075 | 0.77 |  |
| Vanilla flavoring^†^ (Brand: Cape Vanilla) | 0.015 | 0.15 |  |
| Total | **9.740** | **100** |  |
| ^*^*Limnothrissa miodon* and *Stolothrissa tanganicae*.  ^†^non-sweetened. | | | |

**Appendix 2.** Adult Sensory Panel: Evaluation Form

**Appendix 3.** Infant and Young Child (IYC) Sensory Panel: Evaluation Form for Caregiver

**Appendix 4.** List of Acronyms

AI	Adequate Intake

BPA-free	Bisphenol A-free

ComFA+Fish	Complementary Food for Africa+Fish Powder

CHW	community health workers

DRI	dietary reference intake

EFP	extra fish powder

HRPP	Human Research Protection Program

IRB	Institutional Review Board

IPC	Integrated Food Security Phase Classification

IYC	infants and young children

LMIC	low- and middle-income countries

MNP	multiple micronutrient powders

SANAS	South African National Accreditation System-certified laboratory

SSA	sub-Saharan Africa

T1+T2+T3	Time 1+Time 2+Time 3

UNICEF	United Nations Children’s Fund

USAID	United States Agency for International Development

WASH	water, sanitation, and hygiene

WHO	World Health Organization
